# Are Orthodontic Interventions Associated With Headaches in Children and Adolescents? A Systematic Review and Meta‐Analysis

**DOI:** 10.1111/ocr.12911

**Published:** 2025-03-20

**Authors:** Luiz Felipe Tavares, Reid Friesen, Pia Köning, Mareike Neuhaus, Harry von Piekartz, Susan Armijo‐Olivo

**Affiliations:** ^1^ Faculty of Business Management and Social Sciences University of Applied Sciences Osnabrück Osnabruck Germany; ^2^ Postgraduate Program in Physical Therapy (PPGFt) Federal University of São Carlos São Carlos Brazil; ^3^ Faculty of Medicine & Dentistry, Department of Dentistry University of Alberta Edmonton Alberta Canada; ^4^ Faculties of Rehabilitation Medicine, and Medicine and Dentistry University of Alberta Edmonton Alberta Canada

**Keywords:** meta‐analysis, orthodontic appliances, paediatric dentistry, pain

## Abstract

This study aimed to assess the prevalence of headaches in children and adolescents during and after completing orthodontic treatment. This systematic review followed the PRISMA guidelines. An extensive literature search was conducted in the following databases: MEDLINE, Embase, CINAHL, Web of Science, and Cochrane from inception until December 20, 2024. Studies targeting individuals between 7 and 18 years old, diagnosed with malocclusions, and receiving orthodontic treatment were included. A meta‐analysis (odds ratio) was performed considering the number of individuals with and without headache, who did and did not undergo orthodontic treatment. The quality of studies was assessed using the Quality in Prognosis Studies (QUIPS) tool. Finally, the overall certainty of the evidence was assessed by the GRADE approach. Seven studies met the inclusion criteria with a pooled sample size of 1.141 individuals. No statistically significant difference in the prevalence of headache was found between children who received orthodontic intervention compared to no treatment (OR 1.22 [CI 0.78; 1.92]; *p* = 0.38). Children with malocclusion who were treated orthodontically had significantly fewer headaches after treatment compared to untreated children with class II malocclusion (OR 0.42 [CI 0.19; 0.92]; *p* = 0.03). All studies had a high risk of bias, and the certainty of evidence was very low. In summary, no increased prevalence of headaches was detected during or after orthodontic interventions in children and adolescents with malocclusions when compared to those who did not receive orthodontic treatment. Instead, children with malocclusion treated orthodontically had significantly fewer headaches after treatment compared to untreated children with malocclusion. Further research is needed, as the conduct and quality of the existing studies need substantial improvement.

**Trial Registration:** CRD42022340817 (PROSPERO)

## 1 Introduction

Headache is a condition that many people have experienced [1]. Recurrent headaches are common in children and adolescents [2], with approximately 60% experiencing significant headaches and 7.7% to 9.1% migraine [3]. The overall prevalence of primary headache in children and adolescents was recently reported as 62% [4]. Factors such as stress, lack of movement, and muscular tension are mentioned as triggers [5, 6]. Headaches are also often associated with temporomandibular disorders (TMD) and an increased prevalence has been detected [7]. Headaches may limit social activities, physical activity, learning outcomes, and increase school absenteeism, thus impacting quality of life [8]. Migraine is also associated with allergies, sleep disorders, emotional and behavioural problems, and academic performances [4].

In the last decade, the frequency of orthodontic treatment in children and adolescents have increased. Orthodontic issues are typically assessed and addressed during childhood and adolescence. Recent data show a variation between 8% and 33% of the population under 18 years old reporting the use of orthodontics, highlighting the frequent use of these treatments early in life [9, 10, 11, 12, 13]. The American Association of Orthodontists recommends that children have their first orthodontic check‐up by age seven [14]. However, most orthodontic treatments are undertaken between the ages of 10 and 14 when most of the permanent teeth have erupted. Nonetheless, ethnicity plays an important role, whereas orthodontics treatments are more prevalent in non‐Hispanic whites [9]. Moreover, 67.6% of the American population has received some type of orthodontic care [15].

Orthodontic interventions during childhood may be an important contributing factor responsible for forced adaptations of the arthrogenic/myogenic tissues of the temporomandibular joint and craniocervical region [16], some of which may persist beyond the active treatment phase. Such adaptations might stimulate the trigeminal nerve through the pressure exerted on the teeth and surrounding tissues, potentially leading to central sensitisation and linked to headaches [17, 18]. In addition, irradiating pain may arise from adjustments in the temporomandibular joint potentially caused by alterations in jaw alignment during and after orthodontic treatments. Therefore, orthodontic treatment in children and adolescents may play a role in the development of headaches during and after treatment. Likewise, the burden of headache on children and its impact on school performance and quality of life needs further investigation [19].

The relationship between malocclusion, orthodontic treatment, and signs and symptoms of TMD (including headache) has been previously studied [20, 21, 22, 23, 24]. Evidence suggests that severe malocclusions (i.e., misalignment or improper relationship of the teeth and jaws) significantly impact the quality of life of adolescents more negatively compared to mild malocclusions or the absence of malocclusion [25]. Moreover, malocclusion was previously reported as a risk factor for headache in adolescents [26]. Abrahamsson et al. [23] reported a reduction in TMD pain after orthodontic treatment and orthognathic surgery in young adults with different types of malocclusions, while Conti et al. [20] stated no relationship between TMD severity and orthodontic treatment. To the authors’ knowledge, no previous systematic review has specifically assessed the prevalence of headache during and after orthodontic interventions in this population.

Therefore, the objective of this study was to systematically review and assess the prevalence of headaches during and after orthodontic interventions. It was hypothesised that the findings might clarify whether orthodontic interventions may be a contributing factor in paediatric headaches in children and adolescents. Our study question for this review was: Are children and adolescents who undergo or underwent orthodontic interventions more likely to present headaches than those who did not?

## 2 Methods

### 2.1 Data Sources and Searches

This systematic review followed the PRISMA guidelines [27]. The protocol was previously registered in PROSPERO (CRD42022340817). An extensive literature search was conducted in the following databases: MEDLINE, Embase, CINAHL, Web of Science, and Cochrane Library from inception until December 20, 2024. All relevant search terms from previous reviews and newly defined terms by the review team were used. Three main concepts were used to develop the searches: (1) headaches, (2) orthodontic treatments and (3) children and adolescents. No restrictions were placed on the databases in terms of date, language, or publication status. Appendix S1 details the search strategy for each database. To obtain a comprehensive literature overview, the search results were saved in EndNote v20. Subsequently, the results were imported from EndNote to Covidence (www.covidence.org) to facilitate the screening process. After completion, a manual search with citations and references of the included studies was performed on January 6, 2025, via the Web of Science database. In addition, the PROSPERO, ClinicalTrials.gov, and Epistemonikos platforms were searched for possible ongoing studies and related systematic reviews.

### 2.2 Study Selection

This review included any study addressing our research question; however, it was anticipated that most studies would have an observational design (e.g., case–control, cohort, and cross‐sectional). Qualitative studies, commentaries or letters to the editor, conference reports, abstracts without full text, book chapters, and animal studies were excluded. The outcome assessed was the presence of headache. A valid and reliable method to diagnose headache is the International Classification of Headache Disorders (ICHD), which was created by the International Headache Society [28]. The CoCoPop framework [29] was used to guide the selection of studies, according to the following inclusion/exclusion criteria:

#### 2.2.1 Co—Condition

Studies dealing with primary and secondary headaches, according to the International Classification of Headache Disorders (ICHD‐3) [28]. Exclusion criteria were headaches from previous trauma or surgery to the head or neck region, headaches due to tumour diseases, cardiovascular diseases, and neurological diseases. A complete list of headache diagnoses' eligibility is provided in Appendix S2.

#### 2.2.2 Co—Context

The context assessed in this review was orthodontic interventions. All studies including removable or fixed appliances that were used as part of a standard orthodontic treatment with the aim of correcting/improving malocclusion were included. However, orthodontic interventions related to surgery, based on traumatic situations in the past, or related to facial deformity, were excluded. A complete list of orthodontic interventions eligibility is provided in Appendix S3.

#### 2.2.3 Pop—Population

Studies considering the following subjects were included: (1) individuals from 7 to 18 years old, diagnosed with dental malocclusions who were receiving orthodontic treatment, (2) individuals with normal occlusion (i.e., ideal or functional occlusion) and with dental malocclusions who did not receive orthodontic treatment, (3) or both (1 and 2). No limitations were made in relation to gender, ethnicity, and country of residence.

### 2.3 Data Screening

Title and abstract screening was performed by two independent reviewers after the removal of duplicates. Subsequently, the full‐text screening was conducted by two independent reviewers and included studies were selected after consensus. In case of disagreement between the reviewers, the senior author made a final decision. The Covidence (www.covidence.org) platform was used to screen studies.

### 2.4 Data Extraction

Data extraction was initially performed independently by two reviewers and entered directly into an electronic form created in Microsoft Excel, which was previously tested. A third reviewer checked the extracted information from each study. All reviewers who performed the data extraction received the same training and guidance. The training was conducted in detail using one study, and each of the studies was reviewed by all reviewers together and discussed in a group session to ensure consistency in the data extraction.

The data extracted for the assessment were based on features that included information regarding study characteristics, population, treatment, headache characteristics, results summaries, data analyses, conclusions, limitations, and comments and recommendations. The number of individuals (events) reporting headache (before, during, and after orthodontic treatment, when applicable) and the total sample number were used for the odds ratio calculation. Authors were contacted for any missing or unreported data.

### 2.5 Risk of Bias

The risk of bias (RoB) assessments were done independently by two reviewers using the QUIPS tool [30], which has been commonly used for determining the RoB of observational studies. The senior author was consulted in case of disagreements. It contains six domains: study participation, study attrition, prognostic factor measurement, outcome measurement, study confounding, and statistical analysis and reporting. Each domain can be divided into subcategories, which can be rated as yes, partial, no, uncertain, or not applicable. After each subcategory was rated, the final RoB was rated as high (if the study was rated high in at least one domain), moderate (if the study was rated unclear in at least one domain, and the other domains were low) or low (if the study was rated as low risk in all individual domains) according to the guidelines provided by Hayden et al. [30].

### 2.6 Data Synthesis

Data synthesis was done narratively and quantitatively when possible. The number of individuals reporting headache, the total sample size, and the number of individuals who underwent (and did not) orthodontic treatment were considered for the meta‐analysis. For the cohort studies, we chose the most appropriate timepoint that would capture most individuals undergoing orthodontic treatment. When quantitative data were provided, a forest plot was created through RevMan5 software from which the odds ratio (OR), *p* value, and confidence interval (CI) were generated. Heterogeneity was assessed statistically using the *I* ^2^ statistic with *I* ^2^ values of 25%, 50% and 75% representing low, moderate, and high degrees of heterogeneity respectively. The random effects model was chosen for comparison among different studies.

### 2.7 Certainty of the Evidence

The overall quality of the evidence (certainty) was classified according to the Grading of Recommendations, Assessment, Development, and Evaluation (GRADE), using the guidelines provided by Huguet et al. [31] The evidence was classified as high (++++), moderate (+++), low (++) or very low (+). For each domain or risk factor, the following was assessed: (1) phase of investigation, (2) study limitations, (3) inconsistency, (4) indirectness (not generalizable), (5) imprecision (insufficient data), (6) publication bias, (7) effect size and (8) dose effect.

## 3 Results

**FIGURE 1:**
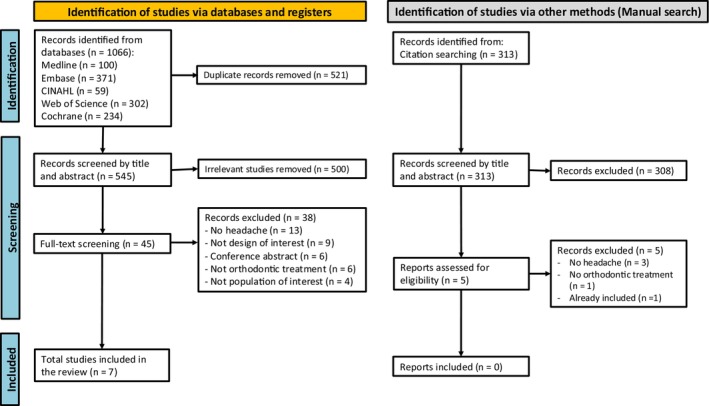
The PRISMA flowchart.

A total of 1.066 studies were imported from the databases. After removing the duplicates, 545 studies were selected for title and abstract screening. Forty‐six studies were read in full, and seven studies were included. Figure 1 describes the PRISMA flowchart with the study steps. Reasons for exclusion are presented in Appendix S4.

General characteristics of included studies are described in Table 1. The included studies were published between 1995 and 2024. Five studies were observational and two were clinical trials. The population age ranged from 7 to 18 years old. Four studies included only fixed appliances as orthodontic interventions. Self‐reported headache was the outcome for five studies, one study included migraine, and one study focused on tension‐type headache. All seven studies assessed headache symptoms after orthodontic treatment, four before treatment, and three during treatment. A detailed description of the included studies is provided in Table 2. No authors were contacted for unreported data.

**TABLE 1:** General characteristics of included studies.

Study characteristic	*n*	Study characteristic	*n*
		**Sex**	
**Country**		Mixed	5
Sweden	2	Female	2
Australia	1		
Finland	1	**Study design**	
Egypt	1	Cohort	3
Bosnia and Herzegovina	1	Case–control	1
Italy	1	Cross‐sectional	1
		Clinical trial	2
**Headache diagnosis**			
TMD‐related headache	3	**Types of orthodontic treatment**	
Tension‐type headache/Migraine	2	Fixed appliances	3
General headache	1	Fixed and removable appliances	1
Primary headache	1	Not specified	1
		Maxillary expansion	2
**Timepoint of assessment**			
Before treatment	4/7	**Language**	
During treatment	3/7	English	7
After treatment	7/7		
		**Funding**	
**Published date**		Not reported	5
Last 5 years	4	Academic	1
Older than 5 years	3	Governmental	1

**TABLE 2:** Detailed description of included studies.

Study details	Sample characteristics	Orthodontic treatment details	Headache diagnosis	Results and conclusions
Egermark and Rönnerman [32] **Study design:** Cohort prospective study **Funding:** Not reported **Setting/Location:** Clinics, Sweden **Objective:** To examine a group undergoing orthodontic treatment with respect to signs and symptoms of TMD and the presence of headache and bruxism as well as prevalence of occlusal interferences	**Age:** 7 to 16 years old **Sex:** male, female **Sample size:** 185 Orthodontic treatment (*n* = 50) vs. control group (*n* = 135) **Occlusion details:** Functional malocclusions, including class II, cross bite, inversion of incisors, scissors bite, and deep bite	Fixed and removable appliances Modified edgewise technique without extraction of premolars (15 patients); with extraction of premolars (32 patients); and removable activators appliances (3 patients) Duration of treatment: 10–27 months	Self‐reported headache diagnosed by a non‐specific questionnaire Duration^a^ of diagnosis: Not reported Timepoint of assesment: before, during and, after treament	The number of participants with headache decreased from 24 to 11 after orthodontic treatment. The reduction was also observed during the active phase of the orthodontic treatment. Malocclusions were largely corrected, and headache prevalence decreased significantly during and after treatment.
Farronato et al. [33] **Study design:** Longitudinal clinical trial **Funding:** Italian Ministry of Health, Current research IRCCS **Setting/Location:** Orthodontic department of the University of Milan, Italy **Objective:** To investigate the role of skeletal modifications due to rapid maxillary expansion on primary headache episodes	**Age:** 7 to 12 years old **Sex:** male, female **Sample size:** 68 Orthodontic treatment (*n* = 68) No comparison group **Occlusion details:** Transverse maxillary deficiency (discrepancy between maxillary and mandibular arches of a minimum of 3 mm and maximum of 6 mm)	Rapid Maxillary Expansion (RME) using a Hyrax expander with bands on upper second deciduous or first permanent molars Activation: 2 turns/day until hypercorrection, followed by a 6‐month stabilisation phase Duration of treatment: At least 6 months of treatment with 6 month stabilisation	Self‐reported headache diagnosed by a non‐specific questionnaire Duration^a^ of diagnosis: Not reported Timepoint of assesment: 30 days before starting treatment and 30 days after treament	The number of headache episodes per month decreased after maxillary expansion. Rapid maxillary expansion significantly reduces primary headache episodes and improves maxillary and nasal dimensions.
Hannan et al. [34] **Study design:** cross‐sectional study **Funding:** Not reported **Setting/Location:** Secondary schools of Sydney, Australia **Objective:** To establish preliminary data for the prevalence of migraine headache among 12–18 year old females who currently have or have previously been fitted with orthodontic braces	**Age:** 12 to 18 years old **Sex:** female **Sample size:** 340 One sample with orthodontic treatment (*n* = 135) and no treatment (*n* = 205) **Occlusion details:** not reported	Fixed appliances; braces Duration of the treatment: 6–110 months	Migraine headaches diagnosed based on:—IHS criteria for paediatric migraine without aura headache must be reported by a duration between 1 and 48 h—previous migraine diagnosis by a health care professional and migraine medication—aura preceding their headache—reporting of pulsating quality and sore eyes—5 attacks or more in the last 12 months Duration^a^ of diagnosis: Not reported Timepoint of assesment: during and after treatment	No difference in prevalence of migraine headache between female adolescents who had brace and those who have not had braces.
Henrikson and Nilner [35] **Study design:** Cohort prospective study **Funding:** Faculty of Odontology at Malmo University, the Swedish Dental Society, and Praktikertjänst AB, Stockholm. **Setting/Location:** Orthodontic Clinics in Public Health Setting, Sweden **Objective:** To study symptoms of TMD and headaches longitudinally in girls with Class II malocclusions receiving orthodontic treatment in comparison with subjects with untreated Class II malocclusions and girls with normal occlusion	**Age:** 11 to 15 years old **Sex:** female **Sample size:** 183 Orthodontic treatment (*n* = 65) vs. normal occlusion (*n* = 60) vs. class II malocclusion (*n* = 58) **Occlusion details:** Class II malocclusion	Fixed appliances – modified straight wire technique to normalise the sagittal, vertical and transversal dental relationships and to eliminate crowding or spacing Duration of the treatment: 14–23 months	Tension‐type headache diagnosed when headaches were reported “once a week or more often” without a migraine diagnosis from a physician. Headache diagnoses were given when patients rated their symptoms “moderate”, “severe” or “very severe” Duration^a^ of diagnosis: Not reported Timepoint of assesment: before and after treament	Participants who received orthodontic treatment for Class II malocclusion reported less symptoms after treatment compared to before, while those with untreated Class II malocclusions reported an increased prevalence of headaches.
Musanovic et al. [36] **Study design:** Case–control study **Funding:** Not reported **Setting/Location:** Local hospital—Public Institution of Health Care Centre in Fojnica, Bosnia and Herzegovina **Objective:** To determine a correlation between the incidence of signs and symptoms of TMD in children aged 12–18 who do not wear a fixed orthodontic appliance and the incidence of signs and symptoms of TMD in children who wear a fixed orthodontic appliance	**Age:** 12 to 18 years old **Sex:** male, female **Sample size:** 120 Orthodontic treatment (*n* = 60) vs. control group (*n* = 60) **Occlusion details:** 30 boys and 30 girls with different types of malocclusion (classified by Angle) and 30 boys and 30 girls with neutroclusion	Fixed appliances (fixed orthodontic straight wire technique) Duration of the treatment: Not reported	Headache or migraine diagnosed by the Research diagnostic criteria for temporomandibular disorders (RDC/TMD) Duration^a^ of diagnosis: Not reported Timepoint of assesment: after treament (compared to untreated)	Headache was prevalent in 15% of participants who wore a fixed orthodontic appliance and in 16.7% of those who did not wear the appliance. The straight wire technique, as a form of fixed orthodontic therapy, did not show any correlation with the presence of signs and symptoms of TMD and headache.
Myllymaki et al. [37] **Study design:** prospective cohort with longitudinal follow‐up **Funding:** Not reported **Setting/Location:** Elementary schools in Finland **Objective:** To determine the impact of malocclusion and orthodontic treatment on symptoms of TMD (including headache)	**Age:** 12, 15 and 32 years old **Sex:** male, female **Sample size:** 195 One group assessed at 12, 15 and 32 years old **At 12 years old:** 28 orthodontically treated chlildren, 167 not treated **At 15 years old:** 29 orthodontically treated chlildren, 161 not treated **Occlusion details:** The most common malocclusions were crowding, Angle Class II division 1 malocclusion and posterior crossbite	Type not described Duration of the treatment: Not reported	Self reported headache diagnosed by a questionnaire categorised in ‘almost daily’, ‘once a week’, ‘two or three times per month’, ‘rarely’, and ‘never’ Duration^a^ of diagnosis: Not reported Timepoint of assesment: after treament (compared to untreated)	The number of participants with headaches was not correlated with orthodontic treatment. Longitudinal changes in occlusion may have an association with TMD symptoms while orthodontic treatment is not associated with the number of symptoms.
Yacout et al. [38] **Study design:** single‐center randomised clinical trial with two parallel arms **Funding:** Not reported **Setting/Location:** University clinic, Egypt **Objective:** To compare the adolescent patient‐reported experience between a miniscrew‐supported expander activated every other day vs. twice per day related to symptoms of pain, pressure, headache, dizziness, speech difficulty, chewing, and swallowing difficulty	**Age:** 12 to 16 years old **Sex:** male, female **Sample size:** 30 Slow maxillary expansion (SME) (*n* = 15) vs. Rapid maxillary expansion (RME) (*n* = 15) **Occlusion details:** not reported	Slow maxillary expansion (SME) with activation every other day vs. rapid maxillary expansion (RME) with two turns of activation daily Four palatal miniscrews were inserted at the following sites bilaterally: between the first and second premolars and between the second premolar and the first molar. A miniscrew‐supported acrylic expander was fabricated using a 9‐mm expansion screw with four holes accommodating the head of the miniscrews that were subsequently filled with lightcure flowable composite. Expansion was considered sufficient when the transverse maxillary discrepancy measured on digital dental casts was corrected. Duration of the treatment: SME: 58.50 + 7.36 months RME: 16.58 + 2.06 months	Self reported headache diagnosed by a non‐specific questionnaire Duration^a^ of diagnosis: Not reported Timepoint of assesment: before, during, and after treament	Participants in the RME group experienced more pain and chewing difficulty than patients in the SME group. However, no significant differences in headache symptoms was found between SME and RME groups.

Abbreviations: IHS, International Headache Society; PAR, peer assessment rating; RDC, research diagnostic criteria; RME, rapid maxillary expansion; SME, slow maxillary expansion; TMD, temporomandibular disorders.

^a^ Duration of diagnosis (since when participants report having headaches).

### 3.1 Risk of Bias Assessment

**FIGURE 2:**
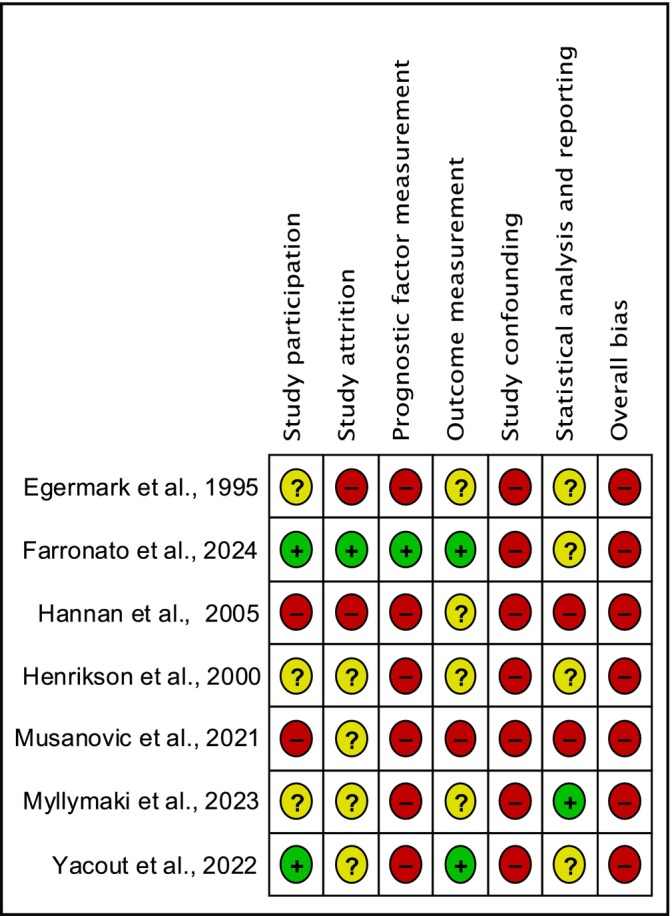
Risk of bias summary.

All seven studies (100%) were rated with a high risk of bias. Main concerns included a lack of clear information regarding the orthodontic interventions, prognostic factor measurement (studies did not perform this analysis based on type of orthodontic treatment or individual characteristics), and control for study confounding (lack of definition on potential variables that might influence the outcome of interest ‐ headache). In addition, some concerns related to study attrition (missing data and dropouts), outcome measurement (lack of valid definitions and tools to determine the diagnosis of headaches) and statistical analysis (inadequate method of analysis and lack of information). Figure 2 shows the risk of bias by individual studies.

### 3.2 Prevalence of Headache During and After Orthodontic Treatment: Narrative Synthesis Analysis

Four of the seven studies reported enough data to be included in the meta‐analysis [32, 35, 36, 37]. The remaining three studies did not provide data to pool their results. Their results will be briefly discussed in this section. Only three studies [32, 34, 38] monitored headache symptoms during orthodontic treatment; all seven studies assessed the prevalence of headache after treatment.

In Hannan et al. [34] study, a total sample of 340 female adolescents from 12 to 18 years old responded to a survey based on the International Headache Society (IHS) criteria. From these, 85.9% reported headaches (*n* = 309). From the 309 adolescents, 23.2% (*n* = 79) had headaches consistent with migraine characteristics. It was also reported that 39.7% (*n* = 135) of the total sample were in current or previous use of braces; however, it was not possible to infer how many of the individuals with headaches had a history of orthodontic treatment, hindering the quantitative analysis. In addition, Yacout et al. [38] did not report the prevalence of headaches. Instead, the intensity of general self‐reported headache (numerical rating scale) was reported as not significantly different between the individuals receiving the rapid maxillary expansion (RME) and slow maxillary expansion (SME). In this study, individuals in the RME group experienced more pain and chewing difficulty than individuals in the SME group. During and after treatment, the RME group reported higher pain and pressure scores than the SME group. However, no significant difference in headache symptoms was reported between the two types of orthodontic treatments (RME vs. SME). In contrast, Farronato et al. [33] reported the number of episodes of headache per month, before and after RME treatment, in 68 children (7 to 12 years old). The number of episodes decreased from 4.74 to 0.59. Furthermore, the four studies [32, 35, 36, 37] included in the meta‐analysis are described below.

### 3.3 Meta‐Analysis: Prevalence of Headache and Orthodontic Treatment

#### 3.3.1 Self‐Reported Weekly Headache Prevalence: Orthodontic Treatment vs. no Orthodontic Treatment in Children and Adolescents With Class II Malocclusion

**FIGURE 3:**
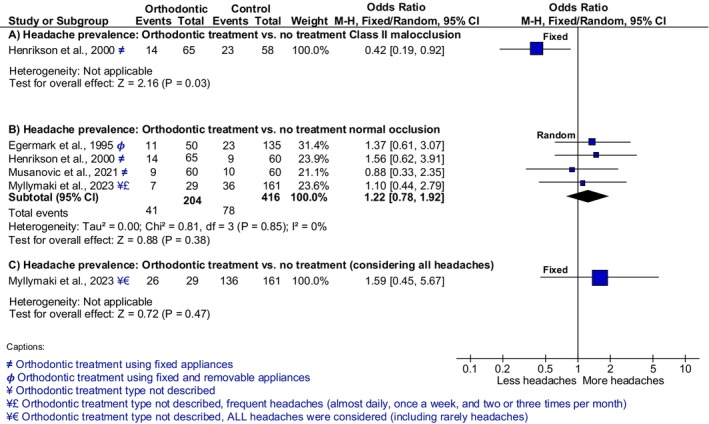
Comparison of headache prevalence between children and adolescents who were and were not orthodontically treated.

Henrikson and Nilner [35] compared three groups to determine the prevalence of TMD and headaches in female children and adolescents before and after treatment with: (1) class II malocclusion who were orthodontically treated; (2) who did not present malocclusion and thus did not receive orthodontic treatment; and (3) with untreated class II malocclusion. In this study, headache diagnosis included a mixed diagnosis between tension‐type headache (diagnosed when headaches were reported weekly in subjects without a migraine diagnosis from a physician) and migraine. Nevertheless, it was not clearly reported how many individuals had each specific diagnosis. From a total of 65 individuals in the orthodontic group, 14 (22%) suffered from headaches, whereas in the class II malocclusion group without orthodontic treatment, 23 (40%) of 58 individuals reported headaches. Based on this analysis, female children and adolescents with class II malocclusion treated orthodontically suffered significantly fewer headaches than female children and adolescents with untreated malocclusion (OR 0.42 [CI 0.19; 0.92] *p* = 0.03; Figure 3, analysis A). Based on that, children and adolescents with a malocclusion who were orthodontically treated had 58% lower odds (0.42 times the odds) of suffering from headaches than children and adolescents with a malocclusion who were not orthodontically treated (Figure 3; comparison A). The results of this comparison are based on a very low certainty of the evidence according to our GRADE assessment (Table 3).

**TABLE 3:** Certainty of the evidence by the GRADE assessment.

Certainty assessment							No of patients		Effect		Certainty
No of studies	Phase of investigation	Study limitations	Inconsistency	Indirectness	Imprecision	Other considerations	Ortho	Nothing	Relative (95% CI)	Absolute (95% CI)	
**Headache prevalence—orthodontic treatment versus no orthodontic treatment (class II malocclusion)**											
1 (Henrikson and Nilner [35])	Phase I non‐randomised studies	Very serious^a^	Not applicable	Not serious	Serious^b^	None	14/65 (21.5%)	23/58 (39.7%)	OR 0.42 (0.19 to 0.92)	**180 fewer per 1000** (from 286 fewer to 20 fewer)	⨁◯◯◯Very low^a^, ^b^
**Headache prevalence—orthodontic treatment versus no treatment (normal occlusion) for frequent headaches**											
4 (Egermark and Ronnerman [32], Henrikson and Nilner [35], Musanovic et al. [36], Myllymaki et al. [37])	Phase I non‐randomised studies	Very serious^a^	Not serious	Serious^c^	Serious^b^	None	41/204 (20.1%)	78/416 (18.8%)	OR 1.22 (0.78 to 1.92)	**32 more per 1000** (from 35 fewer to 120 more)	⨁◯◯◯ Very low^a^, ^c^, ^d^
**Headache prevalence—orthodontic treatment versus no treatment (All headaches, including rarely headaches)**											
1 (Myllymaki et al. [37])	Phase I non‐randomised studies	Very serious^a^	Not applicable	Not serious	Serious^d^	None	26/136 (19.1%)	136/161 (84.5%)	OR 1.59 (0.45 to 5.67)	**52 more per 1000** (from 135 fewer to 124 more)	⨁◯◯◯ Very low^a^, ^d^

Abbreviations: CI, confidence interval; OR, odds ratio.

^a^ Rated as high risk of bias in the QUIPS assessment.

^b^ Low sample size.

^c^ Only female participants were included in some studies.

^d^ Low sample size; wide confidence interval.

#### 3.3.2 General Headache Prevalence: Orthodontic Treatment (For Different Malocclusions) versus no Treatment in Children and Adolescents With Normal Occlusion (i.e., Ideal or Functional Occlusion)

Four studies [32, 35, 36, 37] were used for this comparison and will be described in more detail below. Egermark and Ronnerman [32] assessed signs and symptoms of TMD (including self‐reported headache) before, during, and after orthodontic treatment (fixed appliances) for functional malocclusions (e.g., class II, cross bite, deep bite) and compared these to a control group using subjects from epidemiological data. Eleven individuals (out of 50; 22%) reported headache after orthodontic treatment. In this study (within‐group analysis), the number of individuals reporting headache decreased when compared before (24/50), during (12/50), and after treatment (11/50). In contrast, control subjects had a lower percentage of headaches (23 out of 135; 17%). Henrikson and Nilner [35] also followed headaches and TMD symptoms for 2 years in adolescent girls with class II malocclusion with orthodontic treatment and compared it to a group of adolescent girls with normal occlusion (i.e., ideal or functional occlusion). In the class II orthodontic group, weekly headache decreased from 26% to 22%. In contrast, weekly headache increased from 13% to 15% in the normal occlusion (i.e., ideal or functional occlusion) group. Musanovic et al. [36] assessed the prevalence of TMD‐related headache and migraine symptoms in adolescents that underwent orthodontic treatment for different types of malocclusion (classified by Angle) with fixed appliances compared with those with normal occlusion (i.e., ideal or functional occlusion) who did not receive orthodontic treatment. The question was targeted based on the previous 6 months before the study, and the report of headache was 9/60 individuals of the orthodontically treated group and 10/60 of the untreated group who had normal occlusion (i.e., ideal or functional occlusion). Myllymaki et al. [37] performed a 20‐year prospective study, with assessments at 12, 15, and 32 years old. For our analysis, the 15‐year‐old timepoint was chosen as most representative of the high number of orthodontic treatments for adolescents. In this age group, the orthodontically treated individuals (class II, crowding, posterior crossbite) reported a prevalence of TMD‐related headache of 89.7% (headache daily, once a week, two or three times per month, and rarely). As for the untreated group, the number was 83.7% for the same categories. A sensitive analysis for the reporting of headache of this study is performed and described further (Figure 3; comparison C).

When pooling the studies and comparing children and adolescents treated with fixed appliances with untreated children and adolescents with normal occlusion (i.e., ideal or functional occlusion), three studies [32, 35, 37] favoured more headaches after orthodontic treatment and one study favoured fewer headaches [36] (Figure 3; comparison B). However, all studies had confidence intervals crossing the 1, and the meta‐analysis reported no statistically significant difference in headache prevalence between individuals treated or not treated orthodontically (OR 1.22 [CI 0.78; 1.92]; *p* = 0.38; Figure 3, comparison B). The OR indicates the probability of suffering from headaches after orthodontic treatment in childhood/adolescence was 1.22 times the odds higher than in the control group who were children/adolescents with normal occlusion (i.e., ideal or functional occlusion) that did not require orthodontic treatment (Figure 3; comparison B). The results of this comparison are based on a very low certainty of the evidence according to our GRADE assessment (Table 3).

Furthermore, a sensitivity analysis was performed for the study of Myllymaki et al. [37] (Figure 3; comparisons B and C, highlighted with ¥ symbol). In this study, the self‐reported headache was divided into categories (almost daily, once a week, two or three times per month, rarely, and never). For comparison B, “rarely” and “never” were excluded from the total number of headaches (events). As for comparison C, all categories were summed up (except “never”). This latter comparison showed no difference in the results, with a slight increase in the odds ratio for this specific study (OR 1.59 [CI 0.45; 5.67]; *p* = 0.47; Figure 3; comparison C).

### 3.4 Certainty of the Evidence

The overall certainty of the evidence was very low and is described in Table 3. The combination of high risk of bias, indirectness, and imprecision ultimately resulted in the overall certainty being rated as very low. Study limitations were a significant concern, as most of the included studies were non‐randomised and exhibited a high risk of bias, according to the QUIPS assessment tool. This introduced the potential for confounding variables to affect the outcomes.

## 4 Discussion

### 4.1 Summary of the Results

The estimated overall prevalence of headache in children and adolescents was based on 620 individuals (only four of the seven studies reported enough data). The prevalence of headache was 20.09% after orthodontic treatment and 18.75% for the untreated children and adolescents. However, the meta‐analysis indicated that the prevalence of headaches was not higher in children and adolescents after orthodontic treatment compared to those who did not receive treatment. In addition, qualitatively, the three studies that assessed headache symptoms during active treatment did not report an increase. Therefore, it does not support our hypothesis that orthodontic interventions could be a contributing factor for headaches during or after treatment in children and adolescents. This conclusion is drawn with a high risk of bias and very low certainty of evidence. Due to the limited number of studies assessing the relationship between malocclusion‐related orthodontic interventions and headaches, the results should be interpreted with caution.

A pooled comparison between orthodontic treatment and no orthodontic treatment (in individuals with normal occlusion) was possible among four studies [32, 35, 36, 37]. The overall prevalence of headache was similar between comparisons, with a relatively high prevalence of headache independent of orthodontic treatments. Therefore, orthodontic treatment was not associated with an increased prevalence of headaches based on the analysed studies. The study by Henrikson and Nilner [35] included girls with normal occlusion (i.e., ideal or functional occlusion) and class II malocclusions. The prevalence of headache was lower in the group that did not receive orthodontic treatment and had normal occlusion (25%), followed by the group that was orthodontically treated with malocclusion class II (33.8%), and the non‐treated group with malocclusion class II (69%). The authors attributed the difference between groups to an improved dental occlusion and occlusal stability in the orthodontic group and to individual fluctuations of reported symptoms.

The studies involved different age groups, sample sizes, orthodontic treatments implemented, different headache definitions as well as different comparisons, which contributed to the high clinical heterogeneity. For instance, Henrikson and Nilner [35] focused on girls aged 11–15 years, whereas Hannan [34] studied females aged 12–18 years, and Myllymäki et al. [37] included individuals from age 12 to adulthood. The outcomes measured also varied, from TMD symptoms and headache prevalence to broader assessments of dental health and malocclusion changes. This variability in outcome measures further complicated direct comparisons across studies and firm conclusions.

### 4.2 Orthodontic Treatment Heterogeneity

The heterogeneity of orthodontic treatments and follow‐up durations in the studies is evident, reflecting the complexity and individualised nature of orthodontic care. The differences in treatment types, durations, and follow‐up periods made it challenging to draw uniform conclusions about the effects of orthodontic interventions on headaches.

Regarding treatment heterogeneity, Henrikson and Nilner [35] included various treatment modalities such as fixed appliances and Class II malocclusion corrections. Elastics and extraoral traction devices, such as headgear, were utilised in some individuals. However, the data analysis did not differentiate between these specific treatment types and their respective outcomes. The treatment duration ranged from 14 to 23 months, depending on individual treatment needs. Egermark and Ronnerman [32] studied treatments involving fixed appliances with and without extractions and removable appliances like activators. The active treatment phase varied from 10 to 27 months. Myllymäki et al. [37] assessed different malocclusions, including crowding, Angle Class II division 1 malocclusion, and posterior crossbite, with treatments at various stages. This study's heterogeneity extends to the types of malocclusions treated and the timing of interventions.

Follow‐up periods ranged significantly among the studies, from a few months to several decades. Henrikson and Nilner [35] conducted a two‐year follow‐up, while Myllymäki et al. [37] extended follow‐ups up to 20 years. Egermark and Ronnerman [32] included assessments before, during, and after the treatment phase, capturing both short‐term and intermediate‐term effects. Hannan [34] focused on a one‐time assessment of headache prevalence in relation to orthodontic treatment duration, providing a snapshot rather than a longitudinal perspective. This finding also suggests that, in addition to the variability in the indication, content, duration, method, and starting age of orthodontic treatment, no clear guidelines for orthodontic treatment that may influence the outcome of this study are reported.

### 4.3 Comparison With Previous Reviews

No previous systematic review has assessed the prevalence of headache in children and adolescents during and after orthodontic treatment. Therefore, our results could only be compared with similar conditions, such as TMD. According to the review of Mohlin et al. [39], malocclusions and orthodontic treatment were not associated with TMD. Another systematic review [40] found that individuals with malocclusion after orthodontic surgery had a similar incidence of TMD to the control group without malocclusion and without treatment. These results from previous reviews support our findings. Individuals without malocclusion have the same chances of developing headaches as individuals with orthodontically treated malocclusion. Interestingly, individuals with malocclusion who are treated orthodontically seem to have less frequency of headaches than those who were not treated. A few studies highlight a possible association of lifestyle habits [41] (e.g., sleep pattern, meal regularity, screen exposure) and biopsychosocial factors (e.g., emotional status) [42] with frequent or recurring headaches in children and adolescents, although more research is needed for clarification [43].

### 4.4 Certainty of the Evidence

The certainty of the evidence was rated as very low; thus, indicating ambiguity regarding the prevalence of headache after orthodontic treatments. Main factors contributing to these results include a high risk of bias and imprecision. In the assessment of the studies using the QUIPS tool, all studies showed a high risk of bias. In terms of content, all studies have neither a baseline nor statistical models. A further limitation is that possible confounding factors remained unmentioned in all studies. Indirectness also contributed to the downgrading of the evidence, particularly in studies like Egermark and Ronnerman [32], which included only female individuals, limiting the generalisability of the findings. Additionally, imprecision was a critical issue, with small sample sizes leading to wide confidence intervals in studies such as Myllymaki et al. [37], making it difficult to draw robust conclusions.

### 4.5 Strengths and Limitations

A few limitations can be mentioned. Heterogeneity among studies individuals (e.g., only female individuals or mixed) and orthodontic treatment, and the inclusion of old studies (e.g., from 1995 to 2005) with probable outdated criteria for diagnosing headaches. The included studies in this systematic review used questionnaires that are neither valid nor reliable enough to adequately assess the presence of headaches (i.e., outcome). However, literature searches were conducted without limitation related to language and the year of publication (from 1984 to 2024). Also, no study had to be excluded due to lack of availability, and the inclusion criteria were broad, allowing for the identification of all possible matching studies.

### 4.6 Implications for Research and Clinical Practice

The odds ratio analysis suggests that children and adolescents orthodontically treated and untreated have similar odds of suffering from headaches. In addition, children and adolescents with a malocclusion who were orthodontically treated had 58% lower odds (0.42 times the odds) of suffering from headaches than children and adolescents with a malocclusion who were not orthodontically treated; however, this result is based on one study [35]. For children and adolescents who were treated with a fixed orthodontic appliance, it can be assumed that any headaches that may occur are less likely to have been caused by the orthodontic treatment. However, these results should be viewed with caution, as further studies are needed. There is little information on dosage, length, and type (e.g., active or passive applications) of orthodontic interventions. The diagnosis of headache should also be more specific, including a diagnostic classification using the latest version of the ICHD criteria [28]. Further research should perform longer follow‐ups with pain behaviour, function, and quality of life using measurements like the paediatric migraine disability assessment (MIDAS), craniofacial pain and disability inventory (CF‐PDI), or the short‐form quality of life (SF‐12). It would also be pertinent to investigate further whether headaches arise during orthodontic procedures.

## 5 Conclusion

The general prevalence of headache did not increase during or after orthodontic treatment in children and adolescents. In addition, children and adolescents with normal occlusion (i.e., ideal or functional occlusion) and whose malocclusion has been orthodontically treated reported a similar prevalence of headaches. Class II malocclusion might be associated with a higher headache prevalence, highlighting the importance of assessing malocclusion in children and monitoring its influence on associated symptoms. Long‐term prospective studies are needed to provide robust evidence and a comprehensive understanding of headache outcomes during and after orthodontic treatment.

## Ethics Statement

This study did not involve the collection of primary data from human participants or animals.

## Conflicts of Interest

The authors declare no conflicts of interest.

## Supporting information


Appendix S1.
Appendix S2.
Appendix S3.
Appendix S4.

## Data Availability

All data supporting this systematic review are derived from publicly available studies. References to these studies are included in the manuscript.
