# Supplementary material for: Are Orthodontic Interventions Associated With Headaches in Children and Adolescents? A Systematic Review and Meta‐Analysis
Source: Orthod Craniofac Res. 2025 Mar 20;28(4):656–69. doi: 10.1111/ocr.12911 (PMC12233058; doi:10.1111/ocr.12911)
Supplement: Supplementary file 1 — Appendix S1. Appendix S2. Appendix S3. Appendix S4. [file OCR-28-656-s001.docx]

Appendix 1 Search strategies.

Ovid MEDLINE(R) ALL <1946– December 18, 2024>.

Date searched: December 19, 2024.

Results: 100.

1 exp Headache/32815

2 exp Post-Traumatic Headache/ or exp Headache/ or exp Headache Disorders, Secondary/ or exp. Cluster Headache/ or exp Headache Disorders, Primary/ or headache*.mp. or exp Headache Disorders/138119

3 ((migraine* or cervicogenic or tension) adj3 headache*).mp. [mp = title, book title, abstract, original title, name of substance word, subject heading word, floating sub-heading word, keyword heading word, organism supplementary concept word, protocol supplementary concept word, rare disease supplementary concept word, unique identifier, synonyms, population supplementary concept word, anatomy supplementary concept word] 14 891

4 (cluster headache* or cephalgi* or primary headache* or cephalodyni* or cranial pain* or head ach*).mp. [mp = title, book title, abstract, original title, name of substance word, subject heading word, floating sub-heading word, keyword heading word, organism supplementary concept word, protocol supplementary concept word, rare disease supplementary concept word, unique identifier, synonyms, population supplementary concept word, anatomy supplementary concept word] 7771

5 ((sick or primary or tension or stress) adj3 headache).mp. 8149

6 (sick headache* or primary headache* or primary cephalalgia* or tension headache* or stress headache* or histamine cephalagia* or megrim or migrainous or cephalalgia* or cephalalgy).mp. 7036

7 exp *migraine disorders/ or exp *alice in wonderland syndrome/ or exp *migraine with aura/ or exp *migraine without aura/ or exp *ophthalmoplegic migraine/28574

8 migraine disorders.mp. 31447

9 exp *trigeminal autonomic cephalalgias/ or exp *cluster headache/ or exp *paroxysmal hemicrania/ or exp *sunct syndrome/2891

10 1 or 2 or 3 or 4 or 5 or 6 or 7 or 8 or 9 138748

11 orthodontics.mp. or exp Orthodontics, Corrective/ or exp Orthodontics/ or exp Orthodontics, Interceptive/62791

12 Orthodontics/8994

13 exp Orthodontic Retainers/ or exp. Orthodontic Appliances, Fixed/ or exp Orthodontic Wires/ or exp Orthodontic Appliances/ or orthodontic*.mp or exp Orthodontic Appliances, Removable/ or exp. Orthodontic Appliance Design/ or exp Orthodontic Anchorage Procedures/ or exp Orthodontic Brackets/68795

14 (((((functional class II or functional class III or orthopaedic class II or orthopedic class III) adj3 appliance) or functional appliance or braces or frankel or frankel-2 or frankel-3 or bionator or twin block or forsus or herbst or delaire mask or facial mask or jasper jumper or headgear or mandibular headgear or chin cup or mandibular advance* or mara or maa or mentonera or mascara traccion frontal or activador clase II or activador clase III) adj3 appliance) or functional appliance or braces).mp. [mp = title, book title, abstract, original title, name of substance word, subject heading word, floating sub-heading word, keyword heading word, organism supplementary concept word, protocol supplementary concept word, rare disease supplementary concept word, unique identifier, synonyms, population supplementary concept word, anatomy supplementary concept word] 9330

15 11 or 12 or 13 or 14 84751

16 children.mp. or exp Child/2608443

17 exp. Dental Care for Children/ or children*.mp. or exp Adult Children/ or exp Disabled Children/1353262

18 (children or teenagers or youth or child* or schoolchild* or adolescen* or boy or boys or girl or girls or youth or youths or teen or teens or teenager* or puberty).mp. [mp = title, book title, abstract, original title, name of substance word, subject heading word, floating sub-heading word, keyword heading word, organism supplementary concept word, protocol supplementary concept word, rare disease supplementary concept word, unique identifier, synonyms, population supplementary concept word, anatomy supplementary concept word] 4204978

19 (child or child* or kid or kids or puberty or puberty or pubescen* or prepubescen*or prepuberty* or teen* or juvenile* or preadolesc* or adolescent or adolesc* or young adultor girl*or boy*).mp. [mp = title, book title, abstract, original title, name of substance word, subject heading word, floating sub-heading word, keyword heading word, organism supplementary concept word, protocol supplementary concept word, rare disease supplementary concept word, unique identifier, synonyms, population supplementary concept word, anatomy supplementary concept word] 4222150

20 16 or 17 or 18 or 19 4276318

21 10 and 15 and 20 100

Embase <1974 to 2024 December 18, 2024>.

Date searched: December 19, 2024.

Results: 371.

1 exp Headache/285478

2 exp Post-Traumatic Headache/ or exp Headache/ or exp Headache Disorders, Secondary/ or exp. Cluster Headache/ or exp Headache Disorders, Primary/ or headache*.mp. or exp Headache Disorders/415678

3 ((migraine* or cervicogenic or tension) adj3 headache*).mp. [mp = title, abstract, heading word, drug trade name, original title, device manufacturer, drug manufacturer, device trade name, keyword heading word, floating subheading word, candidate term word] 25201

4 (cluster headache* or cephalgi* or primary headache* or cephalodyni* or cranial pain* or head ach*).mp. [mp = title, abstract, heading word, drug trade name, original title, device manufacturer, drug manufacturer, device trade name, keyword heading word, floating subheading word, candidate term word] 13364

5 ((sick or primary or tension or stress) adj3 headache).mp. 15 151

6 (sick headache* or primary headache* or primary cephalalgia* or tension headache* or stress headache* or histamine cephalagia* or megrim or migrainous or cephalalgia* or cephalalgy).mp. 17425

7 exp *migraine disorders/ or exp *alice in wonderland syndrome/ or exp *migraine with aura/ or exp *migraine without aura/ or exp *ophthalmoplegic migraine/45126

8 migraine disorders.mp. 892

9 exp. *trigeminal autonomic cephalalgias/ or exp *cluster headache/ or exp *paroxysmal hemicrania/ or exp *sunct syndrome/4685

10 1 or 2 or 3 or 4 or 5 or 6 or 7 or 8 or 9 416348

11 orthodontics.mp. or exp Orthodontics, Corrective/ or exp Orthodontics/ or exp. Orthodontics, Interceptive/38051

12 Orthodontics/33097

13 exp. Orthodontic Retainers/ or exp. Orthodontic Appliances, Fixed/ or exp Orthodontic Wires/ or exp Orthodontic Appliances/ or orthodontic*.mp or exp Orthodontic Appliances, Removable/ or exp. Orthodontic Appliance Design/ or exp Orthodontic Anchorage Procedures/ or exp. Orthodontic Brackets/66601

14 (((((functional class II or functional class III or orthopaedic class II or orthopedic class III) adj3 appliance) or functional appliance or braces or frankel or frankel-2 or frankel-3 or bionator or twin block or forsus or herbst or delaire mask or facial mask or jasper jumper or headgear or mandibular headgear or chin cup or mandibular advance* or mara or maa or mentonera or mascara traccion frontal or activador clase II or activador clase III) adj3 appliance) or functional appliance or braces).mp. [mp = title, abstract, heading word, drug trade name, original title, device manufacturer, drug manufacturer, device trade name, keyword heading word, floating subheading word, candidate term word] 4850

15 11 or 12 or 13 or 14 69747

16 children.mp. or exp Child/3488776

17 exp. Dental Care for Children/ or children*.mp. or exp Adult Children/ or exp. Disabled Children/ 1866564

18 (children or teenagers or youth or child* or schoolchild* or adolescen* or boy or boys or girl or girls or youth or youths or teen or teens or teenager* or puberty).mp. [mp = title, abstract, heading word, drug trade name, original title, device manufacturer, drug manufacturer, device trade name, keyword heading word, floating subheading word, candidate term word] 4205145

19 (child or child* or kid or kids or puberty or puberty or pubescen* or prepubescen*or prepuberty* or teen* or juvenile* or preadolesc* or adolescent or adolesc* or young adultor girl*or boy*).mp. [mp = title, abstract, heading word, drug trade name, original title, device manufacturer, drug manufacturer, device trade name, keyword heading word, floating subheading word, candidate term word] 4 232 284

20 16 or 17 or 18 or 19 5006609

21 10 and 15 and 20 350

22 exp. postural headache/ or exp chronic cluster headache/ or exp primary headache/ or exp tension headache/ or exp headache/ or exp. chronic daily headache/ or exp episodic cluster headache/ or exp secondary headache/ or exp “headache and facial pain”/ or exp chronic tension headache/ or headache.mp. or exp thunderclap headache/ or exp cluster headache/ or exp episodic tension headache/ or exp new daily persistent headache/410228

23 orthodontics.mp. or exp orthodontics/38051

24 exp. orthodontic procedure/ or exp orthodontic anchorage/ or orthodontic*.mp. 66203

25 children.mp. or exp child/3488776

26 exp. child/ or child*.mp. 3859155

27 15 or 23 or 24 69747

28 20 or 25 or 26 5006609

29 10 or 22 416348

30 27 and 28 and 29 350

CINAHL Plus with Full Text (EBSCOhost Interface).

Date searched: December 19, 2024.

Results: 59.

S1 (MH “Headache+”) OR “headache” OR (MH “Tension Headache”) OR (MH “Rebound Headache”) OR (MH “Headache, Secondary+”) OR (MH “Headache, Primary+”) OR (MH “Cluster Headache”)

S2 (migraine* or cervicogenic headache or (tension adj3 headache*) or cluster headache* or cephalgi* or primary headache* or cephalodyni* or cranial pain* or head ach*

S3 Headaches or headache disorders

S4 ‘headache and facial pain’/de OR ‘chronic daily headache’/exp OR ‘exertional headache’/exp OR ‘migraine’/exp OR ‘posttraumatic headache’/exp OR ‘postural headache’/exp OR ‘primary headache’/exp OR ‘secondary headache’/exp OR ‘sinus headache’/exp OR ‘stabbing headache’/exp OR ‘tension headache’/exp OR ‘thunderclap headache’/exp OR ‘trigeminal autonomic cephalalgia’/exp OR ‘vascular headache’/exp OR cephalodyni*:ti,ab,kw OR ‘cranial pain*’:ti,ab,kw OR cephalalgi*:ti,ab,kw OR cephalgi*:ti,ab,kw OR headach*:ti,ab,kw OR ‘head ach*’:ti,ab,kw OR migrain*:ti,ab,kw

S5 ‘headache and facial pain’/de OR ‘chronic daily headache’/exp OR ‘exertional headache’/exp OR ‘migraine’/exp. OR ‘posttraumatic headache’/exp OR ‘postural headache’/exp OR ‘primary headache’/exp OR ‘secondary headache’/exp OR ‘sinus headache’/exp OR ‘stabbing headache’/exp OR ‘tension headache’/exp OR ‘thunderclap headache’/exp OR ‘trigeminal autonomic cephalalgia’/exp OR ‘vascular headache’/exp. OR cephalodyni*:ti,ab,kw OR ‘cranial pain*’:ti,ab,kw OR cephalalgi*:ti,ab,kw OR cephalgi*:ti,ab,kw OR headach*:ti,ab,kw OR ‘head ach*’:ti,ab,kw OR migrain*:ti,ab,kw

S6 headache disorders, primary or tension-type headache or migraine disorders or “cluster headache” or trigeminal autonomic cephalalgias or headache or “headache disorders” or “sick headache” or “primary headache” or primary cephalalgias or “tension headache” or “stress headache” or “histamine cephalalgia” or megrim or migrainous or cephalalgia or migraine or cephalalgy

S7 headache disorders, primary or tension-type headache or migraine disorders or “cluster headache” or trigeminal autonomic cephalalgias or headache or “headache disorders” or “sick headache” or “primary headache” or primary cephalalgias or “tension headache” or “stress headache” or “histamine cephalalgia” or megrim or migrainous or cephalalgia or migraine or cephalalgy

S8 ((sick or primary or tension or stress) adj3 headache).mp.

S9 ((sick or primary or tension or stress) adj3 headache).mp

S10 (sick headache* or primary headache* or primary cephalalgia* or tension headache* or stress headache* or histamine cephalagia* or megrim or migrainous or cephalalgia* or cephalalgy).mp.

S11 exp *migraine disorders/ or exp *alice in wonderland syndrome/ or exp *migraine with aura/ or exp *migraine without aura/ or exp *ophthalmoplegic migraine

S12 migraine disorders*

S12 migraine disorders*

S14 exp *trigeminal autonomic cephalalgias/ or exp *cluster headache/ or exp *paroxysmal hemicrania/ or exp *sunct syndrome/

S15 S1 OR S2 OR S3 OR S4 OR S5 OR S6 OR S7 OR S8 OR S9 OR S10 OR S11 OR S12 OR S13 OR S14

S16 orthodontic*

S17 orthodontics

S18 ((functional class II or functional class III or orthopaedic class II or orthopedic class III) adj3 appliance or functional appliance or braces) or ((frankel or frankel-2 or frankel-3 or bionator or twin block or forsus or herbst or delaire mask or facial mask or jasper jumper or headgear or mandibular headgear or chin cup or mandibular advance* or mara or maa or mentonera or mascara traccion frontal or activador clase II or activador clase III) adj3 appliance or functional appliance or braces

S19 S16 OR S17 OR S18

S20 children

S21 child*

S22 children or teenagers or youth or child* or schoolchild* or adolescen* or boy or boys or girl or girls or youth or youths or teen or teens or teenager* or puberty

S23 child or child* or kid or kids or puberty or puberty or pubescen* or prepubescen*or prepuberty* or teen* or juvenile* or preadolesc* or adolescent or adolesc* or young adultor girl*or boy*

S24 S20 OR S21 OR S22 OR S23

S25 S15 AND S19 AND S24

Web of Science.

Date searched: December 20, 2024.

Results: 302.

1: TS = (headache) Results: 211150

2: TS = (migraine disorders) Results: 63650

3: TS = (cervicogenic headache) Results: 1596

4: AK = (sick OR primary OR tension OR stress NEAR headache) Results: 336134

5: AK = headaches OR AK = headache disorders Results: 22552

6: AK = (migraine*OR cervicogenic headache OR tension headache OR cluster headache* OR primary headache OR cranial pain OR cephalodynia) and Preprint Citation Index (Exclude—Database) Results: 5127

7: AK = (migraine disorders OR alice in wonderland syndrome OR migraine with aura OR migraine without aura OR ophthalmoplegic migraine) and Preprint Citation Index (Exclude—Database) Results: 4074

8: AK = (trigeminal autonomic cephalalgias OR cluster headache OR paroxysmal hemicrania OR sunct syndrome) and Preprint Citation Index (Exclude—Database) Results: 2000

9: AK = (sick headache* OR primary headache* OR primary cephalalgia* OR tension headache* OR stress headache* OR histamine cephalalgia* OR megrim OR migrainous OR cephalalgia* OR cephalalgy) and Preprint Citation Index (Exclude—Database) Results: 4602

10: AK = (headache OR facial pain OR chronic daily headache OR exertional headache OR migraine OR posttraumatic headache OR postural headache OR cluster headache OR paroxysmal hemicrania OR primary headache OR secondary headache OR sinus headache OR stabbing headache OR thunderclap headache OR trigeminal autonomic cephalalgia OR vascular headache) and Preprint Citation Index (Exclude—Database) Results: 41323

11: (#10 OR #9 OR #8 OR #7 OR #6 OR #5 OR #4 OR #3 OR #2 OR #1) Results: 578630

12: TS = (orthodontics*) Results: 42380

13: TS = (orthodontic) Results: 89471

14: AK = (functional class II OR functional class III OR orthopaedic class II OR orthopedic class III NEAR appliance OR functional appliance OR braces) OR AK = (frankel OR frankel-2 OR frankel-3 OR bionator OR twin block OR forsus OR herbst OR delaire mask OR facial mask OR jasper jumper OR headgear OR mandibular headgear OR chin up OR mandibular advance* OR mara OR maa OR mentonera OR mascara traccion frontal OR activador clase II OR activador clase III) and Preprint Citation Index (Exclude—Database) Results: 11241

15: #12 OR #13 OR #14 Results: 116528

16: TS = (children) Results: 5210278

17: AK = (child OR child* OR kid OR kids OR puberty OR pubescen* OR prepubescen* OR prepuberty* OR teen* OR juvenile* OR preadolesc* OR adolescent* OR adolesc* OR young adult girl* OR boy*) Results: 870996

18: AK = (children OR teenagers OR youth OR child* OR schoolchild OR adolescen* OR boy OR boys OR girls OR girl OR youth OR youths OR teen OR teens OR teenager* OR puberty) Results: 887718

19: #16 OR #17 OR #18 Results: 5429553

20: #11 AND #15 AND #19 and Preprint Citation Index (Exclude—Database) Results: 302

COCHRANE Library.

Date searched: December 20, 2024.

Results: 234.

#1 MeSH descriptor: [Headache] explode all trees 3237

#2 exp. Post-Traumatic Headache or exp Headache or exp Headache Disorders, Secondary or exp Cluster Headache or exp Headache Disorders, Primary or headache*.mp. or exp Headache Disorders 1327

#3 migraine* or cervicogenic headache or tension adj3 headache* or cluster headache* or primary headache* or cranial pain or cephalodyni* 25762

#4 Headache or Headache Disorders or cephalodyni* or cranial pain* or cephalalgi* or headach* or head ach* or migrain* 51 337

#5 ‘headache and facial pain’ or ‘chronic daily headache’ or ‘exertional headache’ or ‘migraine’ or ‘posttraumatic headache’ or ‘postural headache’ or ‘primary headache’ or ‘secondary headache’ or ‘sinus headache’ or ‘stabbing headache’ or ‘tension headache’ or ‘thunderclap headache’ or ‘trigeminal autonomic cephalalgia’ or ‘vascular headache’ or cephalodyni* or ‘cranial pain’ or cephalalgi* or cephalgi* or headach* or ‘head ach*’ or migrain* 51 323

#6 exp *trigeminal autonomic cephalalgias or exp *cluster headache or exp *paroxysmal hemicrania or exp *sunct syndrome 547

#7 exp *migraine disorders or exp *alice in wonderland syndrome or exp *migraine with aura or exp *migraine without aura or exp *ophthalmoplegic migraine 176

#8 (sick headache* or primary headache* or primary cephalalgia* or tension headache* or stress headache* or histamine cephalalgia* or megrim or migrainous or cephalalgia* or cephalalgy).mp. 21 503

#9 #1 or #2 or #3 or #4 or #5 or #6 or #7 or #8 71096

#10 MeSH descriptor: [Orthodontics] explode all trees 3840

#11 functional class II or functional class III or orthopaedic class II or orthopedic class III adj3 appliance or functional appliance or braces or frankel or frankel-2 or frankel-3 or bionator or twin block or forsus or herbst or delaire mask or facial mask or jasper jumper or headgear or mandibular headgear or chin up or mandibular advance* or mara or maa or mentonera or mascara traccion frontal or activador clase II or activador clase III adj3 appliance 25023

#12 #10 or #11 28 154

#13 MeSH descriptor: [Child] explode all trees 83722

#14 child or child* or kid or kids or puberty or pubescen* or prepubescen* or prepuberty* or teen* or juvenile* or preadolesc* or adolescent* or adolesc* or young adult girl* or boy* 357654

#15 children or teenagers or youth or child* or schoolchild* or adolescen* or boy or boys or girls or girls or youth or youths or teen or teens or teenager* or puberty 353685

#16 #13 or #14 or #15 359914

#17 #9 and #12 and #16 809

#18 Clinical Trials 234.

Appendix 2 Headaches diagnosis eligibility criteria.

Based on the ICHD-3^1^ definitions, specific primary and secondary types of headaches were included and excluded as follows:

INCLUDED diagnosis:

1. Primary headache disorders

Migraine.

Migraine without aura.

Migraine with aura.

Chronic migraine.

Migraine complications (e.g., status migrainosus, persistent aura without infarction).

Tension-type headache (TTH).

Infrequent episodic TTH.

Frequent episodic TTH.

Chronic TTH.

2. Secondary headache disorders

Headache attributed to disorders of the cranium, neck, eyes, ears, nose, sinuses, or teeth.

Sinusitis-related headache.

Temporomandibular joint (TMJ) headache.

3. Other headache disorders

Other headache disorders.

Headaches not otherwise specified (NOS).

EXCLUDED diagnosis:

1. Primary headache disorders

These are headaches not caused by another condition.

Trigeminal autonomic cephalalgias (TACs).

Cluster headache.

Paroxysmal hemicrania.

Short-lasting unilateral neuralgiform headache attacks (SUNCT/SUNA).

Hemicrania continua.

Other primary headaches.

Primary stabbing headache.

Primary cough headache.

Primary exercise headache.

Primary headache associated with sexual activity.

Hypnic headache.

New daily persistent headache (NDPH).

2. Secondary headache disorders

Headache attributed to trauma or injury.

Post-traumatic headache (acute or chronic).

Headache attributed to vascular disorders.

Headache attributed to ischaemic stroke or transient ischaemic attack (TIA).

Headache attributed to intracranial haemorrhage.

Cervical artery dissection.

Headache attributed to non-vascular intracranial disorders.

Idiopathic intracranial hypertension (IIH).

Intracranial hypotension.

Headache attributed to substance use or withdrawal.

Medication-overuse headache.

Headache due to acute substance use (e.g., alcohol-induced headache).

Headache attributed to infection.

Meningitis or encephalitis headache.

Systemic infection headache (e.g., flu-related headache).

Headache attributed to homeostasis disruption.

Headache due to high blood pressure.

Hypoxia-induced headache.

3. Other headache disorders

Cranial neuralgias and central causes of facial pain.

Trigeminal neuralgia.

Glossopharyngeal neuralgia.

Reference

1. ICHD-3. Headache Classification Committee of the International Headache Society (IHS) The International Classification of Headache Disorders, 3rd edition. Cephalalgia. 2018;38(1):1–211. doi:[10.1177/0333102417738202](http://dx.doi.org/10.1177/0333102417738202)

Appendix 3 Orthodontic treatments and their indications.

Removable orthodontic treatments.

| **Orthodontic treatment** | **Type of treatment** | **Indications/conditions treated** |
| --- | --- | --- |
| **Hawley retainers** | Removable retainer | Used post-treatment to maintain tooth alignment. |
| **Essix retainers** | Removable retainer | Transparent retainers used for post-treatment retention of teeth. |
| **Clear aligners** | Removable appliance (e.g., Invisalign) | Utilised for mild to moderate malocclusions such as crowding, spacing, and minor bite corrections. |
| **Functional appliances** | Removable (e.g., twin block, bionator) | Indicated for jaw growth modification, Class II correction, and mandibular advancement. |
| **Expansion appliances** | Removable (e.g., Slow Maxillary expansion—SME) | Used for transverse maxillary expansion, particularly in younger patients with crowding or posterior crossbite. |

**Fixed orthodontic treatments**

| **Orthodontic treatment** | **Type of treatment** | **Indications/conditions treated** |
| --- | --- | --- |
| **Metal braces** | Fixed braces | Indicated for the correction of various malocclusions, including Class I, II, and III, crowding, and spacing. |
| **Ceramic braces** | Fixed braces | An aesthetic alternative to metal braces, used to correct a wide range of malocclusions. |
| **Lingual braces** | Fixed braces (Behind the teeth) | Indicated for patients who want discrete treatment; corrects most malocclusions. |
| **Self-ligating Braces** | Fixed braces (no elastic ties) | Used to reduce friction during treatment; indicated for a variety of malocclusions. |
| **Rapid maxillary expansion (RME)** | Fixed expansion appliance | Used for transverse maxillary deficiency and correction of posterior cross bite in children. |
| **pendulum appliance** | Fixed appliance | Used to move molars posteriorly for space management in crowding cases. |
| **Nance appliance** | Fixed appliance | Used to maintain space in the upper arch after orthodontic extractions. |
| **Transpalatal arch (TPA)** | Fixed appliance | Employed for upper molar anchorage and maintaining arch form during treatment. |
| **Headgear** | Fixed (cervical-pull, High-pull, reverse-pull) | Used for Class II correction or maxillary restriction in skeletal discrepancies. |
| **Class II elastics** | Fixed elastics | Used in conjunction with fixed braces to correct overjet and Class II malocclusion. |
| **Class III elastics** | Fixed elastics | Utilised for correcting underbite and Class III malocclusions. |
| **Temporary anchorage devices** | Semi-fixed appliance | Provide additional anchorage for specific tooth movements such as molar distalisation or intrusion without affecting adjacent teeth. |

References

Proffit, W. R., Fields, H. W., Larson, B., & Sarver, D. M. (2019). Contemporary orthodontics (6th ed.). Mosby Elsevier.

Appendix 4 Reasons for exclusion table.

| First Author / Year | Full title | Reason |
| --- | --- | --- |
| Abreu 2018 [1] | Orthodontics in Children and Impact of Malocclusion on Adolescents' Quality of Life | No headache |
| Alpaydin 2024 [2] | Do symptoms and signs of temporomandibular disorders have an association with breathing pattern: a cross-sectional study on Turkish children and adolescents | No orthodontic treatment |
| Barone 1997 [3] | Craniomandibular disorders and orthodontic treatment need in children | No orthodontic treatment |
| Bergius 2008 [4] | Prediction of prolonged pain experiences during orthodontic treatment | No headache |
| Botos 2016 [5] | Interdisciplinary osteopathic—orthodontic treatment of patients with posture alterations: Case presentation | Not design of interest |
| Buchanan 2005 [6] | Malocclusion as a risk factor in the aetiology of headache in children | No orthodontic treatment/Dissertation |
| Campos 2013 [7] | The influence of patient's motivation on reported pain during orthodontic treatment | No headache |
| Carra 2010 [8] | Teeth clenching and sleep bruxism in 7–17 years old population: Risk factors for temporomandibular, sleep and breathing disorders | Conference abstract |
| Carra 2013 [9] | Sleep bruxism, snoring, and headaches in adolescents: short-term effects of a mandibular advancement appliance | Conference abstract |
| Carra 2011 [10] | Sleep bruxism and headache in adolescents: An experimental trial with a mandibular advancement appliance (preliminary data) | Conference abstract |
| Carra 2012 [11] | Symptomatic sleep bruxism in adolescents: An experimental trial with a mandibular advancement appliance | Not design of interest |
| Conti 2003 [12] | Relationship between signs and symptoms of temporomandibular disorders and orthodontic treatment: a cross-sectional study | No headache |
| Drobek 2015 [13] | Prevalence of tension type headache in children and adolescents with temporomandibular disorders heading for orthodontic treatment | Conference abstract |
| ElNaghy 2023 [14] | Do patient-reported outcomes of miniscrew-supported maxillary expansion in adolescent patients differ between slow and rapid activation protocol? | Not design of interest |
| Feldmann 2007 [15] | Reliability of a questionnaire assessing experiences of adolescents in orthodontic treatment | Not design of interest |
| Firestone 1999 [16] | Patients' anticipation of pain and pain-related side effects, and their perception of pain as a result of orthodontic treatment with fixed appliances | Not design of interest |
| Gianni 1995 [17] | [Primary headache and orthognathorhinodontia in childhood] | Investigation of a special intervention |
| Goldreich 1994 [18] | The effect of pain from orthodontic arch wire adjustment on masseter muscle electromyographic activity | No headache |
| Hasan 2019 [19] | Oral health problems in patients receiving orthodontic treatment-a survey | No headache |
| Heredia-Rizo 2014 [20] | Masticatory mechanosensitivity, mouth opening and impact of headache in subjects with a history of orthodontics use: a cross-sectional study | Not adolescents |
| Jones 1992 [21] | The pain and discomfort experienced during orthodontic treatment: a randomised controlled clinical trial of two initial aligning arch wires | No headache |
| Lambourne 2007 [22] | Malocclusion as a risk factor in the aetiology of headaches in children and adolescents | No orthodontic treatment |
| Martins Junior 2011 [23] | Attitudes of a group of Brazilian orthodontists towards the diagnosis and management of primary headache (migraine): an electronic-based survey | Not design of interest |
| Martins 2021 [24] | Impact of wearing palatal expanders on the quality of life of children aged 8 to 10 years | No headache |
| Mohlin 1991 [25] | A survey of craniomandibular disorders in 1000 12-year-olds—study design and base-line data in a follow-up-study | No orthodontic treatment |
| Musanovic 2021 [26] | Prevalence of TMD among children provided with fixed orthodontic treatment | No headache |
| Needleman 2000 [27] | Reports of pain by children undergoing rapid palatal expansion | No headache |
| Ohmori 2022 [28] | Associations among temporomandibular disorder, headache, shoulder pain, craniofacial morphology, and premature contacts in female children and adolescents with malocclusion | Not design of interest |
| Pilleyj 1992 [29] | A survey of craniomandibular disorders in 800 15-year-olds a follow-up study of children with malocclusion | Not possible to isolate the treatment group |
| Saccomanno 2021 [30] | The relationship between tmj symptoms and orthodontic treatments: A survey on 236 orthodontic patients | Not adolescents |
| Saloom 2018 [31] | The effect of obesity on orofacial pain during early orthodontic treatment with fixed appliances: a prospective cohort study | No headache |
| Simunovic 2024 [32] | The Role of Malocclusion and Oral Parafunctions in Predicting Signs and Symptoms of Temporomandibular Disorders-A Cross-Sectional Study | No orthodontic treatment |
| Sletten 2015 [33] | The effect of specially designed and managed occlusal devices on patient symptoms and pain: a cohort study | Not adolescents |
| SoaresBonato 2022 [34] | Tooth movement, orofacial pain, and leptin, interleukin-1beta, and tumour necrosis factor-alpha levels in obese adolescents | No headache |
| Thavarajah 2015 [35] | Analysis of adverse events with use of orthodontic sequential aligners as reported in the manufacturer and user facility device experience database | Not adolescents |
| Valkeapaa 2024 [36] | Sleep and other indicators of quality of life during orthodontic treatment with fixed or removable appliance | Conference abstract |
| Yuan 2024 [37] | Comparison of functional impairments and discomfort with bonded labial and lingual orthodontic appliances—a questionnaire survey | No headache |
| Zaverdinos 2019 [38] | Prevalence of bruxism and mouth breathing in craniofacial patients | Conference abstract |

References

1. Abreu LG. Orthodontics in Children and Impact of Malocclusion on Adolescents' Quality of Life. Pediatr Clin North Am. 2018;65:995−+, 1006.

2. Alpaydin MT, Alpaydin T, Torul D. Do symptoms and signs of temporomandibular disorders have an association with breathing pattern: a cross-sectional study on Turkish children and adolescents. BMC Oral Health [Internet]. United Kingdom; 2024;24:721. Available from: <https://login.ezproxy.library.ualberta.ca/login?url=http://ovidsp.ovid.com/ovidweb.cgi?T=JS&CSC=Y&NEWS=N&PAGE=fulltext&D=emexb&DO=10.1186%2Fs12903-024-04482-5https://resolver.library.ualberta.ca/resolver?sid=OVID:embase&id=pmid:38914975&id=doi:10.1186%2Fs> NS -

3. Barone A, Sbordone L, Ramaglia L. Craniomandibular disorders and orthodontic treatment need in children. J Oral Rehabil [Internet]. 1997;24:2–7. Available from: <https://login.ezproxy.library.ualberta.ca/login?url=http://ovidsp.ovid.com/ovidweb.cgi?T=JS&CSC=Y&NEWS=N&PAGE=fulltext&D=med4&AN=9049912>

4. Bergius, M, Broberg, AG, Hakeberg, M, Berggren, U. Prediction of prolonged pain experiences during orthodontic treatment. Am J Orthod Dentofac Orthop [Internet]. United States: Mosby Inc. (11830 Westline Industrial Drive, St. Louis MO 63146, United States); 2008;133:339.e1-339.e8. Available from: NS -

5. Botos, A, Chelaru, H, Mesaros, A, Irimie, A, Grecu, A, Dudea, D. Interdisciplinary osteopathic - Orthodontic treatment of patients with posture alterations: Case presentation. Clujul Med [Internet]. 2016;89(Supplem:S182. Available from: <http://www.clujulmedical.umfcluj.ro/public/public/Supplements/2016-supplement-4.zip>

6. Buchanan, WC . Malocclusion as a risk factor in the aetiology of headache in children [Internet]. 2005. Available from: NS -

7. Campos, MJ da S, Vitral, RWF. The influence of patient's motivation on reported pain during orthodontic treatment. Dental Press J Orthod [Internet]. 2013;18:80–5. Available from: NS - NS -

8. Carra, M. . Teeth clenching and sleep bruxism in 7-17 years old population: Risk factors for temporomandibular, sleep and breathing disorders. Sleep Breath [Internet]. 19th Annual Meeting of the American Academy of Dental Sleep Medicine, San Antonio, Texas, USA, 4–6 June 2010; 2010. p. 274–5. Available from: NS -

9. CarraMCHuynhNTEl-KhatibHRemiseCLavigneGJSleep bruxism, snoring, and headaches in adolescents: short-term effects of a mandibular advancement applianceSleep Med20131465661. Available from: https://login.ezproxy.library.ualberta.ca/login?url=http://ovidsp.ovid.com/ovidweb.cgi?T=JS&CSC=Y&NEWS=N&PAGE=fulltext&D=med10&AN=23643652710.1016/j.sleep.2013.03.009

10. Carra MC, Huynh NT, Rompre P, Lavigne G. Sleep bruxism and headache in adolescents: An experimental trial with a mandibular advancement appliance (preliminary data). Cephalalgia [Internet]. 2011;1):133. Available from: <https://login.ezproxy.library.ualberta.ca/login?url=http://ovidsp.ovid.com/ovidweb.cgi?T=JS&CSC=Y&NEWS=N&PAGE=fulltext&D=emed12&AN=70625214>

11. Carra, M. Symptomatic sleep bruxism in adolescents: An experimental trial with a mandibular advancement appliance. Sleep [Internet]. Associated Professional Sleep Societies, LLC; 2012;35:A390. Available from: <http://www.journalsleep.org/Resources/Documents/2012abstractsupplement.pdf> NS -

12. Conti, A, Freitas, M Fau - Conti, P, Conti, P Fau - Henriques, J, Henriques, J Fau - Janson, G, Janson, G, Angle, O. Relationship between signs and symptoms of temporomandibular disorders and orthodontic treatment: a cross-sectional study. Angle Orthod. 2003;

13. Drobek. Prevalence of tension type headache in children and adolescents with temporomandibular disorders heading for orthodontic treatment. Cephalalgia [Internet]. SAGE Publications Ltd; 2015. p. 1-296. Available from: NS - This reference is missing a URL or DOI. The "NS -" is likely an indicator of an unavailable link.

14. ElNaghyRAl-QawasmiRHasaninMDo patient-reported outcomes of miniscrew-supported maxillary expansion in adolescent patients differ between slow and rapid activation protocol?Evid Based Dent [Internet]. United Kingdom: NLM (Medline); 202324289. Available from: NS - NS -110.1038/s41432-023-00858-8

15. FeldmannIListTJohnMTBondemarkLReliability of a questionnaire assessing experiences of adolescents in orthodontic treatmentAngle Orthod [Internet]. United States: Angle Orthodontist Research and Education Foundation Inc. (2700 Lake Road Windsor, Huntsville TX 77340–1556, United States);2007773117Available from: http://www.angle.org/pdfserv/i0003-3219-077-02-0311.pdfNS -210.2319/0003-3219(2007)077[0311:roaqae]2.0.co;2

16. FirestoneARScheurerPABurginWBPatients' anticipation of pain and pain-related side effects, and their perception of pain as a result of orthodontic treatment with fixed appliancesEur J Orthod19992138796. Available from: https://login.ezproxy.library.ualberta.ca/login?url=http://ovidsp.ovid.com/ovidweb.cgi?T=JS&CSC=Y&NEWS=N&PAGE=fulltext&D=med4&AN=10502901410.1093/ejo/21.4.387

17. Gianni E, Farronato GP. [Primary headache and orthognathorhinodontia in childhood]. Ital J Neurol Sci [Internet]. 1995;16:57–68. Available from: <https://login.ezproxy.library.ualberta.ca/login?url=http://ovidsp.ovid.com/ovidweb.cgi?T=JS&CSC=Y&NEWS=N&PAGE=fulltext&D=med3&AN=8751190>

18. Goldreich, H, Gazit, E, Lieberman, MA, Rugh, JD. The effect of pain from orthodontic arch wire adjustment on masseter muscle electromyographic activity. Am J Orthod Dentofac Orthop [Internet]. United States; 1994;106:365–70. Available from: NS -

19. Hasan, Kamal; Selvarasu, Kathiravan; Dinesh S. Oral health problems in patients receiving orthodontic treatment-a survey. Drug Invent Today [Internet]. India: JPR Solutions (E-mail: [editorparial@gmail.com](mailto:editorparial@gmail.com)); 2019;11:1598–602. Available from: <http://jprsolutions.info/view_journal_new.php?journal_id=2> NS -

20. Heredia-Rizo AM, Rodriguez-Blanco C, Oliva-Pascual-Vaca A, Torres-Lagares D, Albornoz-Cabello M, Pina-Pozo F, et al. Masticatory mechanosensitivity, mouth opening and impact of headache in subjects with a history of orthodontics use: a cross-sectional study. Eur J Phys Rehabil Med [Internet]. 2014;50:411–8. Available from: <https://login.ezproxy.library.ualberta.ca/login?url=http://ovidsp.ovid.com/ovidweb.cgi?T=JS&CSC=Y&NEWS=N&PAGE=fulltext&D=med11&AN=24963817>

21. M. J.The pain and discomfort experienced during orthodontic treatment: a randomised controlled clinical trial of two initial aligning arch wires. Am J Orthod Dentofacial Orthop [Internet]. United States; 1992;102:373–81. Available from: NS - This appears to be an incomplete reference. The "Available from: NS -" is unclear and requires further investigation.410.1016/0889-5406(92)70054-e

22. Lambourne C, Lampasso J, Buchanan Jr. WC, Dunford R, McCall W. Malocclusion as a risk factor in the aetiology of headaches in children and adolescents. Am J Orthod Dentofac Orthop [Internet]. 2007;132:754–61. Available from: <https://login.ezproxy.library.ualberta.ca/login?url=http://ovidsp.ovid.com/ovidweb.cgi?T=JS&CSC=Y&NEWS=N&PAGE=fulltext&D=med6&AN=18068593>

23. Martins Junior RL, Kerber F de C, Stuginski-Barbosa J. Attitudes of a group of Brazilian orthodontists towards the diagnosis and management of primary headache (migraine): an electronic-based survey. J Appl Oral Sci [Internet]. 2011;19:674–8. Available from: <https://www.scielo.br/j/jaos/a/pWfmzd7RXbTbpSgm7PZfSYg/?lang=en&format=pdf>, 678

24. Martins, MT , De Melo Vitorino, V , Rodrigues, L V , Lages, EMB , Pretti, H , Vale, MP et al Impact of wearing palatal expanders on the quality of life of children aged 8 to 10 years. 2021;21. Available from: <https://www.cochranelibrary.com/central/doi/10.1002/central/CN-02298707/full> NS -

25. Mohlin, B, Pilley, JR, Shaw, WC. A survey of craniomandibular disorders in 1000 12-year-olds. Study design and baseline data in a follow-up study. Eur J Orthod [Internet]. 1991;13:111–23. Available from: NS -

26. Musanovic A, Ajanovic M, Redzepagic Vrazalica L, Kazazic L, Tosum Poskovic S, Mlaco Durek J, et al. Prevalence of TMD among Children Provided with Fixed Orthodontic Treatment. Acta Stomatol Croat [Internet]. 2021;55:159–67. Available from: <https://login.ezproxy.library.ualberta.ca/login?url=http://ovidsp.ovid.com/ovidweb.cgi?T=JS&CSC=Y&NEWS=N&PAGE=fulltext&D=pmnm5&AN=34248149>

27. Needleman, H L, Hoang, C D, Allred, E, Hertzberg, J CB. Reports of pain by children undergoing rapid palatal expansion. Pediatr Dent [Internet]. United States; 2000;22:221–6. Available from: NS - NS -

28. Ohmori, H, Kirimoto, H, Kamaratih, A, Aida, J, Ono, T. Associations among temporomandibular disorder, headache, shoulder pain, craniofacial morphology, and premature contacts in female children and adolescents with malocclusion. Clin Investig Orthod [Internet]. 2022;81:202–8. Available from: NS - Available from: NS -

29. PilleyJRMohlinBShawWCKingdonAA survey of craniomandibular disorders in 800 15-year-olds. A follow-up study of children with malocclusionEur J Orthod19921415261. Available from: NS - NS -210.1093/ejo/14.2.152

30. SaccomannoSLaganaDMastrapasquaRGiancasproSManentiRJSaranSThe relationship between tmj symptoms and orthodontic treatments: A survey on 236 orthodontic patientsJ Biol Regul Homeost Agents2021353 Suppl197204. Available from: <https://www.biolifesas.org/biolife/category/journals/journal-of-biological-regulators-and-homeostatic-agents/>10.23812/21-3supp1-22

31. SaloomHFPapageorgiouSNCarpenterGHCobourneMTThe effect of obesity on orofacial pain during early orthodontic treatment with fixed appliances: a prospective cohort studyEur J Orthod [Internet]. United Kingdom; 2018403439. Available from: NS - NS -410.1093/ejo/cjx064

32. Simunovic, L, Varga, ML, Vranic, DN, Cukovic-Bagic, I, Bergman, L, Mestrovic, S. The Role of Malocclusion and Oral Parafunctions in Predicting Signs and Symptoms of Temporomandibular Disorders-A Cross-Sectional Study. Dent J [Internet]. 2024;12. Available from: NS - NS -

33. Sletten WO, Taylor LP, Goodacre CJ, Dumont TD. The effect of specially designed and managed occlusal devices on patient symptoms and pain: a cohort study. Gen Dent [Internet]. Chicago, Illinois: Academy of General Dentistry; 2015;63:46–53. Available from: <https://login.ezproxy.library.ualberta.ca/login?url=https://search.ebscohost.com/login.aspx?direct=true&db=rzh&AN=103375966&site=ehost-live&scope=site>

34. Soares BonatoRCAbel MapengoMAde Azevedo-SilvaLJJansonGde Carvalho Sales-PeresSHTooth movement, orofacial pain, and leptin, interleukin-1β, and tumour necrosis factor–α levels in obese adolescentsAngle Orthod [Internet]. United States: NLM (Medline); 20229295100. Available from: NS - This appears to be an incomplete reference. The "Available from" field is cut off.110.2319/011321-44.1

35. ThavarajahRThennukondaRAnalysis of adverse events with use of orthodontic sequential aligners as reported in the manufacturer and user facility device experience databaseIndian J Dent Res [Internet]. India; 201526582. Available from: NS - 610.4103/0970-9290.176919

36. Valkeapää W, Arrais de Campos L, Ikävalko T, Peltomäki T. Sleep and other indicators of quality of life during orthodontic treatment with fixed or removable appliance. Sleep Med [Internet]. Netherlands: Elsevier B.V.; 2024;115:S72–3. Available from: <https://login.ezproxy.library.ualberta.ca/login?url=http://ovidsp.ovid.com/ovidweb.cgi?T=JS&CSC=Y&NEWS=N&PAGE=fulltext&D=emexa&DO=10.1016%2Fj.sleep.2023.11.232https://resolver.library.ualberta.ca/resolver?sid=OVID:embase&id=pmid:&id=doi:10.1016%2Fj.sleep>. NS -

37. L. Y, T. D, L. L, N. O, N. Y, et al. Comparison of functional impairments and discomfort with bonded labial and lingual orthodontic appliances - a questionnaire survey. BMC Oral Health [Internet]. United Kingdom; 2024;24:1272. Available from: <https://login.ezproxy.library.ualberta.ca/login?url=http://ovidsp.ovid.com/ovidweb.cgi?T=JS&CSC=Y&NEWS=N&PAGE=fulltext&D=emexb&DO=10.1186%2Fs12903-024-05041-8https://resolver.library.ualberta.ca/resolver?sid=OVID:embase&id=pmid:39449038&id=doi:10.1186%2Fs> NS -

38. Zaverdinos, M, Elshebiny, T, Valiathan, M. Prevalence of bruxism and mouth breathing in craniofacial patients. Cleft Palate Craniofacial J [Internet]. Netherlands: SAGE Publications Ltd; 2019. p. 1-130. Available from: NS - This seems to be an incomplete URL.
